# Biosorption of Pb(II) Ions by *Klebsiella* sp. 3S1 Isolated from a Wastewater Treatment Plant: Kinetics and Mechanisms Studies

**DOI:** 10.1155/2015/719060

**Published:** 2015-10-04

**Authors:** Antonio Jesús Muñoz, Francisco Espínola, Manuel Moya, Encarnación Ruiz

**Affiliations:** Department of Chemical, Environmental and Materials Engineering, University of Jaén, 23071 Jaén, Spain

## Abstract

Lead biosorption by *Klebsiella* sp. 3S1 isolated from a wastewater treatment plant was investigated through a Rotatable Central Composite Experimental Design. The optimisation study indicated the following optimal values of operating variables: 0.4 g/L of biosorbent dosage, pH 5, and 34°C. According to the results of the kinetic studies, the biosorption process can be described by a two-step process, one rapid, almost instantaneous, and one slower, both contributing significantly to the overall biosorption; the model that best fits the experimental results was pseudo-second order. The equilibrium studies showed a maximum lead uptake value of 140.19 mg/g according to the Langmuir model. The mechanism study revealed that lead ions were bioaccumulated into the cytoplasm and adsorbed on the cell surface. The bacterium  *Klebsiella* sp. 3S1 has a good potential in the bioremoval of lead in an inexpensive and effective process.

## 1. Introduction

Biotechnology has a great potential to remove heavy metals using the ability of different bacteria and other microorganisms to capture metal ions, mainly through biosorption. This ability has a high potential for the development of effective and economic processes for heavy metal bioremoval, especially for dilute solutions (<100 mg/L) [1]. Low cost is the most important advantage of biosorption over conventional treatments because biosorbents are usually inexpensive and abundant materials, and biosorption also often offers the advantages of metal recuperation and biomass regeneration [2]. The main mechanisms involved can be cell-surface binding, intracellular bioaccumulation, and extracellular precipitation [3]. The binding of metal on the cell surface can occur with living microorganism or death biomass. Nevertheless, metal bioaccumulation takes place only in living cells.

Microorganisms and other biomass types have the advantage of having components as lipopolysaccharides, proteins, and phospholipids which have many functional groups. These groups confer a negative charge and thus offer the possibility of adding metal cations. This ability is present in all types of biomass, dead or alive. In the case of living microorganisms, other active cellular mechanisms are involved: synthesis of specific enzymes, action of cytoplasmic or membrane proteins, and so forth [4, 5].

In order to improve biosorption effectiveness, the identification of additional microbial strains with high metal uptake capacity and specificity is a key aspect. In this way, the isolation of autochthonous microorganisms from contaminated sites is an interesting option to obtain metal-resistant strains [6–8].

Lead is known for its high environmental impact and toxicity [9]. This metal, along with mercury and cadmium, is considered one of “the big three” heavy metals present in contaminated effluents [2]. The European Directive 2008/105/EC includes lead and its compounds in the list of priority substances in environmental quality standards, establishing a concentration of lead of 0.0072 mg/L as the maximum permissible in the case of surface water.

In this work, the biosorption characteristics of* Klebsiella* sp. 3S1 were investigated. This strain is a bacterium selected from a group of microorganisms isolated from wastewater treatment plants that showed high resistance to several heavy metals, namely, Pb, Zn, and Ag [10]. Kinetic studies were conducted to determine the optimal working time. A Rotatable Central Composite Design (RCCD) with six central points was employed to optimise the operational conditions: sorbent dosage, pH, and temperature. The interactions between the experimental factors were evaluated by Response Surface Methodology (RSM). This technique has proved its effectiveness in optimising the variables that influence the adsorption process and reducing both time and cost [11–13]. Finally, equilibrium studies were performed. Additionally, potential mechanisms of biosorption were studied by FTIR, SEM, and TEM techniques.

## 2. Materials and Methods

### 2.1. Preparation of* Klebsiella* sp. 3S1

Ten strains were selected and identified by molecular techniques in a previous study from wastewater treatment plants [10]. After preliminary biosorption tests, the bacterial strain* Klebsiella* sp. 3S1 was selected for kinetic and equilibrium studies. This isolate showed 98% homology with that of standard* Klebsiella* species based on its 16S-rDNA gene sequence (1476 bp) and was named* Klebsiella* sp. 3S1 (GenBank accession number HE975030).

For the biosorption experiments, the biomass was prepared by incubating the strain in tryptic soy broth (TSB, 30 g/L) medium at 30°C with stirring, after centrifugation at 6000 rpm and washing twice with electrolyte solution. The cells of* Klebsiella* sp. 3S1 used in kinetic studies and the adsorption isotherm were harvested by centrifugation from exponential phase cultures. Then, the cells were resuspended in the lead solution.

### 2.2. FTIR Spectroscopy

The infrared spectra of the biomass samples before and after metal uptake were recorded using a VERTEX 70 (Bruker Corporation) Fourier transform infrared spectrometer operating in the range of 4000–400 cm^−1^. Measurements were performed with lyophilised samples. The samples were measured using Attenuated Total Reflection (ATR). FTIR characterisation was performed to identify characteristic chemical functional groups of the bacterium* Klebsiella* sp. that might be involved in the metal uptake procedure.

### 2.3. FE-SEM-EDX Analysis

Field emission scanning electron microscopy (MERLIN of Carl Zeiss) coupled with energy dispersive X-ray spectroscopy was carried out to characterise the* Klebsiella* sp. 3S1 surface before and after Pb(II) ion uptake. Samples were fixed with a 2.5% glutaraldehyde solution in PBS (pH 7.4), dehydrated with increasing solutions of acetone (50, 70, 90, and 100%), and, finally, dried by critical point drying and metalized with carbon.

### 2.4. HR-TEM-EDX Analysis

High-resolution transmission electron microscopy and energy dispersive X-ray analysis (Philips CM-20) were used to determine the location of the metal within the cell. The samples were fixed, dehydrated, and dried as in the previous case; these were then treated with resin and were finally polymerised.

### 2.5. Batch Biosorption

The test solutions of Pb(II) were prepared using Pb(NO_3_)_2_ and 0.1 M NaCl as an electrolyte. The initial pH was controlled with 0.1 M NaOH or 0.1 M HNO_3_.

The preliminary biosorption tests were performed in Erlenmeyer flasks with the ten strains (Section 2.1). The biosorbents were suspended in 50 mL of Pb(II) solution (25 mg/L) to reach different cell concentrations. The cell/metal suspension was gently agitated (200 rpm) at 30°C in an orbital shaker (CERTOMAT IS, Sartorius). To prevent precipitation of the metal, the lead solution was adjusted to pH 5.0. After 24 h, the metal solution was filtered (pore size 0.22 *μ*m). Finally, the sample was acidified with HNO_3_ and analysed by absorption atomic spectrometry (AAS) using an AANALYST 800 (Perkin Elmer) to know the final metal concentration. The tests for each strain were performed in triplicate.

Isothermal and kinetic studies were conducted as described above. Two kinetics experiments were performed, with two different concentrations of biomass and metal: Experiment number 1: 50 mg/L of Pb(II) and 0.11 g/L of dry biomass and Experiment number 2: 25 mg/L of Pb(II) and 0.28 g/L of dry biomass. The adsorbent was separated from the solution by centrifugation at predetermined time intervals (5–3600 min), with two flasks at each time point. Then, the residual lead concentration was analysed by AAS. The biosorption isotherm was obtained for a constant biomass concentration (0.52 g/L) and different concentrations of metal at 25°C.

In all cases, the amount of metal ions biosorbed per unit mass of dry biosorbent (lead biosorption capacity) was determined according to the following equation:

(1) $$\begin{array}{c} q=\frac{\left(C_{i}-C_{f}\right)V}{m}, \end{array}$$

where *q* is the biosorption capacity (mg metal/g of the dry biosorbent); *C*_*i*_ and *C*_*f*_ are the initial and final metal concentrations, respectively; *V* is the liquid volume (0.05 L); and *m* is the amount of the biosorbent sample on a dry basis (g).

### 2.6. Experimental Design and Statistical Analysis

A Rotatable Central Composite Design (RCCD) with six central points was performed to evaluate the relationship between obtained results and experimental factors as well as optimise the working conditions. Biosorbent dosage (*B*), pH, and temperature (*T*) were chosen as independent variables, and the equilibrium biosorption capacity (*q*_*e*_) was chosen as the dependent output response variable. The lead concentration used in all cases was 100 mg/L.

According to the RCCD, the total number of experimental combinations is 2^*k*^ + 2*k* + *n*_0_, where *k* is the number of independent variables and *n*_0_ is the number of repetitions of the experiments at the centre point. The independent variables (factors), experimental range, and levels for lead removal are given in Table 1. The experimental design incorporated 20 experimental points, including six replicates at the central point.

The overall quadratic equation for biosorption capacity was

(2) qe=β0+β1B+β2pH+β3T+β11B2+β22pH2+β33T2+β12BpH+β13BT+β23pHT,

where *q*_*e*_ is equilibrium biosorption capacity (mg metal/g of the dry biosorbent); *B* is the biosorbent dosage; *T* is the temperature (°C); *β*_0_ is the interception coefficient; *β*_1_, *β*_2_, and *β*_3_ are the linear terms; *β*_11_, *β*_22_, and *β*_33_ are the quadratic terms; and *β*_12_, *β*_13_, and *β*_23_ are the interaction terms.

The response data were analysed by parameters obtained from the analysis of variance (ANOVA) using Design-Expert program, 8.0.7.1 version. The statistical significance was fixed at 5% probability level (*p* value = 0.05).

### 2.7. Modelling of Uptake Kinetics

To know the biosorption mechanisms and speed of the process, it is important to study the mass transfer and chemical reactions. To do this, the experimental data have been adjusted to several kinetic models. In most cases, it is assumed that adsorption is controlled by chemical reaction and not by diffusion, which contributes to the mechanical agitation. However, we have tested three kinetic models of chemical reaction control and one of intraparticle diffusion. The kinetic models tested are shown in Table 2. To adjust the experimental data to the kinetic models, we used the IBM SPSS Statistics software, 19 version (SPSS Inc. and IBM Company).

### 2.8. Modelling of the Biosorption Isotherm

Several adsorption isotherms have been tested to fit experimental data. The most widely used among them are the Langmuir and Freundlich models; other well-known models are the Sips and Redlich-Peterson equations (Table 3). Adsorption isotherms originally used for gas phase adsorption can be readily adapted to correlate adsorption equilibrium in heavy metals biosorption. The Langmuir model establishes a relationship between the amount of gas adsorbed on a surface and the pressure of gas and assumes monolayer coverage of the adsorbate over a homogeneous adsorbent surface. The Freundlich model is an empirical equation, which assumes that as the adsorbate concentration in solution increases it also increases on the adsorbent surface. This model can be applied to nonideal sorption on heterogeneous surfaces as well as to multilayer sorption. The Sips model is also called the Langmuir-Freundlich isotherm; this isotherm model incorporates features of both the Langmuir and Freundlich isotherms and may be used to represent adsorption equilibrium over a wide concentration range. The Redlich-Peterson model is another empirical equation that can represent adsorption equilibrium over a wide concentration range. When *β* = 1, it effectively reduces to a Langmuir isotherm, and when *β* = 0, it obeys Henry's law. To adjust the experimental data to kinetic models, IBM SPSS Statistics software described above was employed.

## 3. Results and Discussion

### 3.1. Preliminary Biosorption Tests

Ten strains isolated from wastewater treatment plants showed resistance to lead in our previous study [10]. Preliminary biosorption tests were performed on these strains to select the most appropriate one.  Table 4 shows the results obtained, which range from 45 to 104 mg/g for fungi, 22 to 38 mg/g for yeast, and 78 to 90 mg/g for bacteria. The best results are obtained with the fungus* Trichosporon* sp. 1L1 and the bacterium* Klebsiella* sp. 3S1.

To choose the most suitable strain for heavy metals biosorption, other aspects of interest should be taken into account. It is very interesting to support microbial biomass in low cost inert solids. Therefore, microorganisms with greater ability to form biofilms are always the most appropriate [31]. This was the reason why that* Klebsiella* sp. 3S1 was chosen.

### 3.2. FTIR Spectroscopic Study

**Figure 1:**
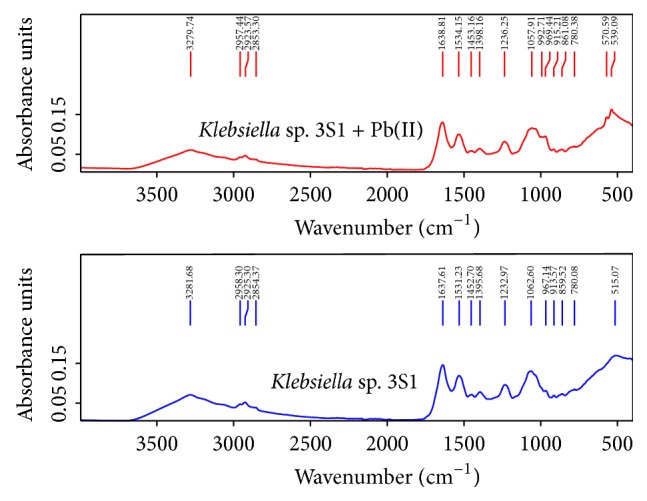
FTIR spectra of* Klebsiella* sp. 3S1 before and after Pb(II) biosorption.

FTIR analysis was performed to identify the main functional groups and to study their evolution during biosorption process. This technique has proved effective in obtaining structural information on metal-microbe bonds [32]. The results for* Klebsiella* sp. 3S1 showed the presence of the following functional groups: amino, carbonyl, carboxylic, hydroxyl, and phosphate.  Table 5 shows band assignments and the typical functional groups present in this bacterium before and after Pb(II) uptake [33–35]. The IR spectra of metal-free and metal-loaded biomass showed differences in the functional groups (Figure 1). After contact with the metal solution, the spectra obtained show a shift of the frequency band and the appearance of new peaks [36]; furthermore, a decrease in band intensity was observed. These changes indicate that the functional groups are involved in the process of adsorption. Moreover, it is important to note the appearance of a peak at 1038 cm^−1^; this peak occurs after contact with the metal solution, and this shift is typical of the complexation of phosphate or carboxyl groups by coordination with metal ions [37, 38]. Finally, two new peaks were observed in the region with low wave numbers (under 800 cm^−1^); these peaks (571 and 539 cm^−1^) could be attributed to the interaction between the metal ions and N-containing bioligands [39, 40].

### 3.3. Chemical and SEM Analysis

**Figure 2:**
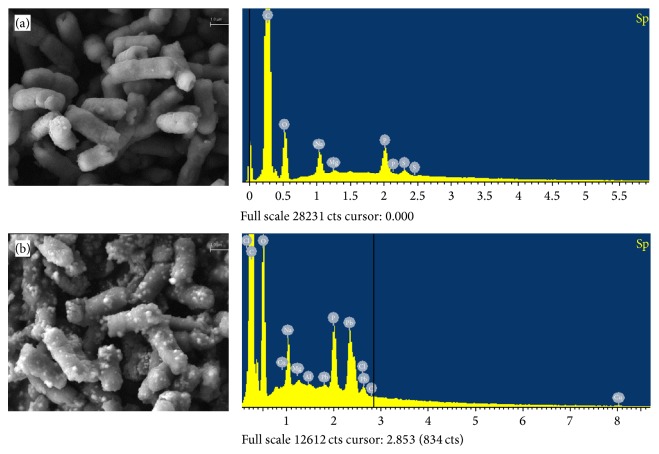
SEM-EDX analysis of* Klebsiella* sp. 3S1 before (a) and after Pb(II) biosorption (b).

Figure 2 shows micrographs and EDX spectra obtained after and before the biosorption process. These micrographs show changes in the cell morphology of* Klebsiella* sp. 3S1; there is also a large presence of bright particles on the surface of the bacteria treated with lead. EDX analysis supported this observation, confirming that the metal remained adsorbed by the entire cell surface. Other authors have obtained similar results with different microorganisms [39, 41].

### 3.4. Chemical and TEM Analysis

**Figure 3:**
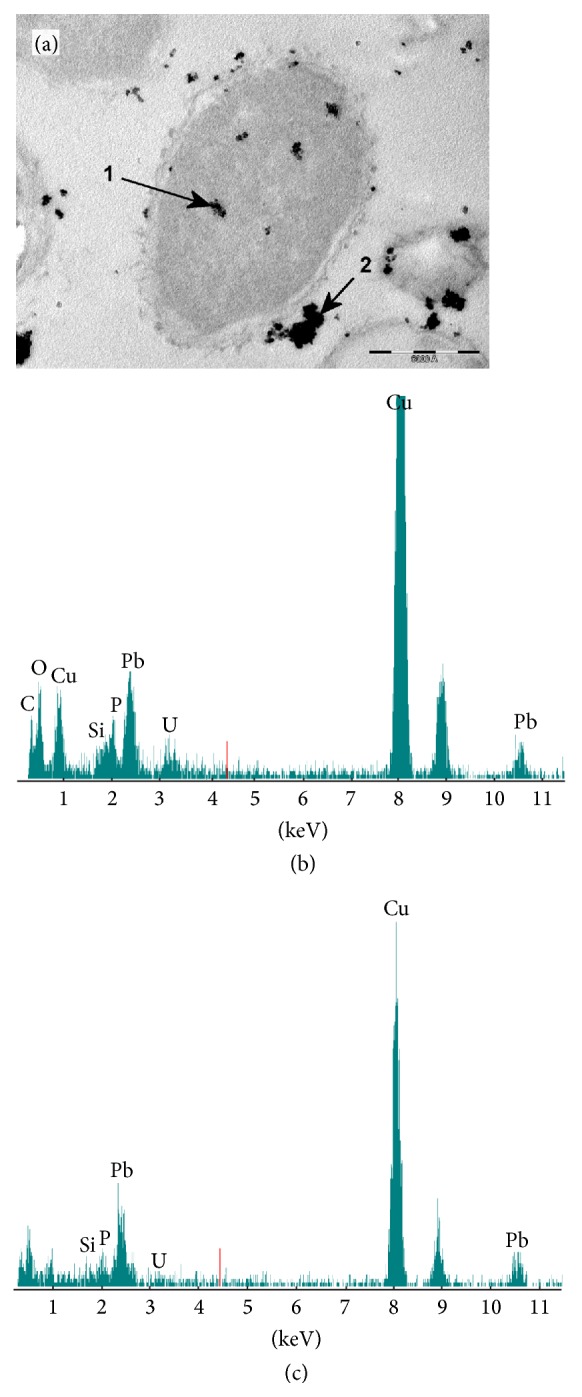
Transmission electron micrographs of a thin section of* Klebsiella* sp. 3S1 and the location of fixed lead (a). Energy dispersive X-ray spectra of the intracellular accumulation of lead (b) acquired from the region indicated by arrow 1 in (a) and the surface biosorption (c) acquired from the region indicated by arrow 1 in (a).

The location of the metal within the cell was evaluated using HR-TEM-EDX technique. The micrographs obtained together with the corresponding EDX microanalysis (Figure 3) show that the metal is largely fixed at the cell surface. However, in some cases, lead accumulation also appears within the cytoplasm, which could indicate the presence of a bioaccumulation mechanism; some authors have identified this capacity in different microorganisms [42]. Perdrial et al. [43] localised lead accumulations associated with polyphosphate bodies in bacteria isolated from the environment.

### 3.5. Uptake Kinetics

**Figure 4:**
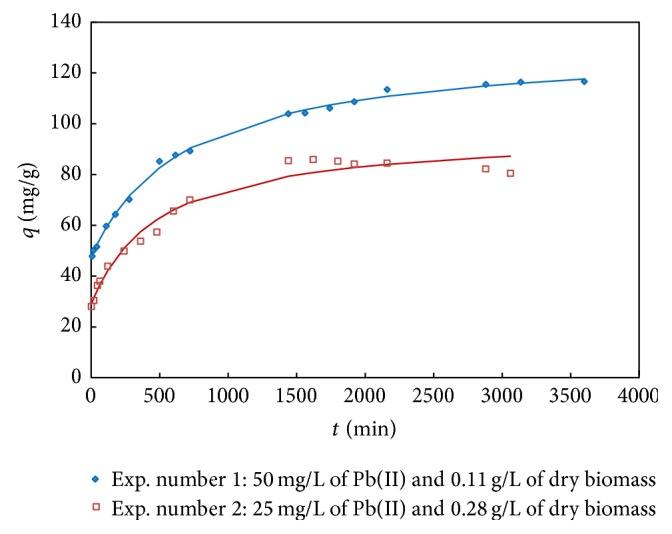
Experimental data and curves corresponding to the model that best fits the experimental results, pseudo-second order with boundary conditions: *q* = *q*_*i*_ at *t* = 0 and *q* = *q* at *t* = *t*.

Sorption kinetics is important because it describes the solute uptake, which also controls the residence time of the metal ions at the solid-solution interface.  Figure 4 shows the results of two biosorption experiments. The observed values are the average of two replicates. From the figure, it can be observed that the biosorption occurs in two phases: first a very fast initial rate within the first 5 min and then a slow attainment of equilibrium within 1 to 2 days, both of which contribute to the total metal biosorption. This finding suggests that adsorption of lead may occur by two mechanisms: cell-surface binding and intracellular accumulation. To contemplate both mechanisms, we used different boundary conditions. Ho et al. [44] considered an imaginary negative time when the rate of sorption is infinity, in the same case.

Kinetic behavior during adsorption has been studied by numerous models [45]. They have attempted to quantitatively describe the process. However, they present limitations that can be solved with theoretical assumptions and specific experimental conditions. The models that best fit our experimental data were pseudo-first and second order. To fit models to data, we used linear and nonlinear regression (always getting the best results from nonlinear regressions) and used different boundary conditions; namely, *q* = *q*_*i*_ at *t* = 0 and *q* = *q* at *t* = *t*, where *q*_*i*_ corresponds to a quick, practically instantaneous biosorption at the beginning of the experiment. The use of the latter boundary conditions is a major difference from what is found in the literature, as the typical boundary condition is *q* = 0 at *t* = 0.

Table 6 shows the values of the parameters that best fit the models provided. The higher correlation coefficients confirm that pseudo-second-order kinetics is the most suitable to represent the biosorption data. This fact also supports the assumption of the model, namely, that adsorption is due to chemisorption. Our results are similar to those obtained by other authors with different microorganisms [14, 21].  Figure 4 also shows the curves corresponding to the model that best fits the experimental results so that the experimental data can easily be compared with the model predictions.

### 3.6. Response Surface Modelling and Optimum Biosorption Conditions

A Rotatable Central Composite Design was performed. Table 1 shows the values of the operating variables, that is, biosorbent dosage (*B*), pH and temperature (*T*), and the biosorption capacity (*q*_*e*_) response. The results were fit to a quadratic equation for biosorption capacity, (2), by using the Design-Expert statistical package. The final equation in terms of actual factors is shown below:

(3) qe=−483.84+245.13B+193.33pH+0.65726T−105.22B2−18.598pH2+0.071415T2−13.477BpH−2.7294BT.

Analysis of variance (ANOVA) was performed to determine the influence of the significant factors involved in the biosorption capacity.  Table 7 shows the variability of the factors and their interactions. The effect of a factor can be defined as the response variation produced by a change in the factor level, where only terms found to be statistically significant were included. In this case, seven effects showed *p* values below 0.05. This indicates that they are significantly different from zero with a 95% confidence level and one required to support hierarchy.

The model is significant (*F*-value = 437.66) and the lack of fit is not significant (*F*-value = 2.42). Therefore, it can be stated that the model can predict biosorption capacity as an equation of the three studied factors. The *R*-Squared suggests that the model explains 99.74% of the variability in the response.

**Figure 5:**
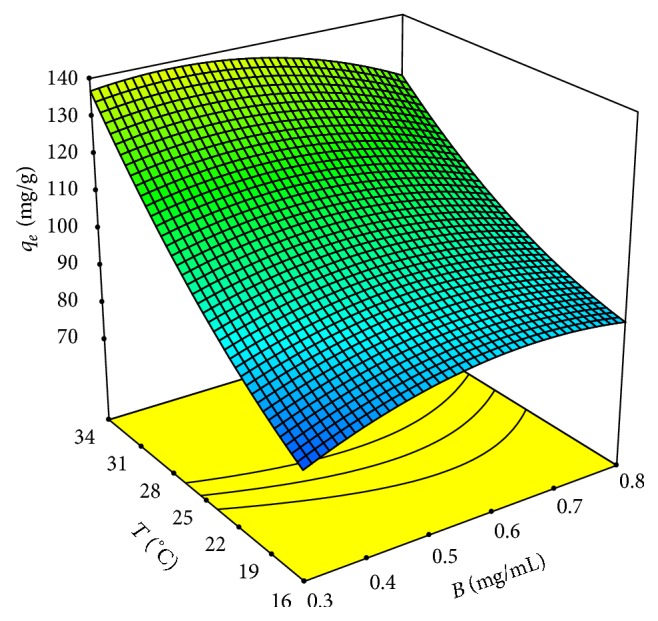
Response surface plot for biosorption of Pb(II) by* Klebsiella* sp. 3S1 showing the interactive effect of temperature (*T*) and biosorbent dosage (*B*) (pH, 4.75, and initial lead concentration, 100 mg/L).

The perturbation plots were obtained to study the effects of several factors. These plots showed that temperature has the greatest influence on Pb(II) removal efficiency, and the biosorbent dosage exerts less influence. This is shown in Figure 5, with the response surface corresponding to pH 4.75. Moreover, this figure also shows that *q*_*e*_ increases with biosorbent dosage for low values of temperature, whereas if the temperature values are high, the biosorbent dosage influence is negative; this is justified by the interaction between the biosorbent dosage and temperature.

**Figure 6:**
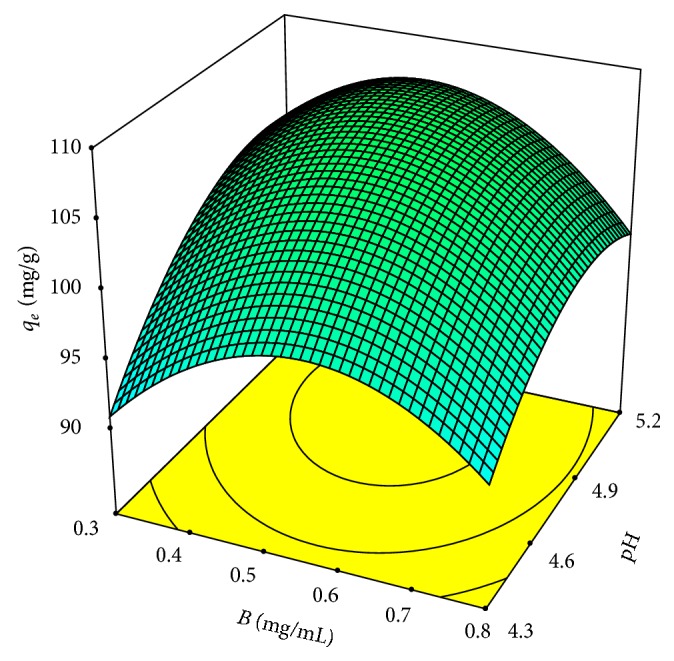
Response surface plot for biosorption of Pb(II) by* Klebsiella* sp. 3S1 showing the interactive effect of biosorbent dosage (*B*) and pH (temperature, 25°C, and initial lead concentration, 100 mg/L).

The empirical model given as (3) was also optimised: the maximum *q*_*e*_, 139.96 mg/g, was mathematically located at 34°C, pH 5.05, and biosorbent dosage 0.40 mg/mL. However, the adjusted model also allows different operational conditions, for example, a temperature of 25°C, which is closer to room temperature (Figure 6).

### 3.7. Biosorption Equilibrium

**Figure 7:**
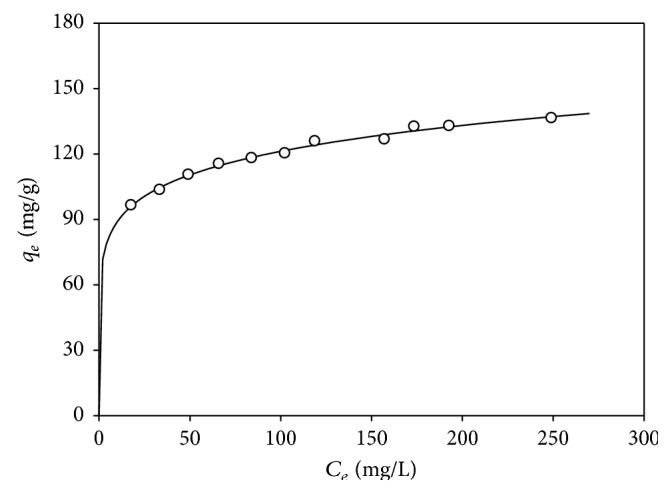
Biosorption equilibrium data and Freundlich isotherm for* Klebsiella* sp. 3S1.

Equilibrium data were obtained experimentally using different initial concentrations of Pb(II) between 50 and 320 mg/L and a constant biomass concentration (0.52 g/L), at 25°C and pH 5 (Figure 7). These are the optimum conditions according to the obtained model, (3), given a temperature of 25°C, which was chosen because it is more like environmental conditions.

Four isotherm equations have been examined in the present study. All parameters were adjusted by nonlinear regression.  Table 8 shows the results obtained. The biosorption isotherms for Pb(II) ion uptake by* Klebsiella* sp. 3S1 were found to be appropriate for all predictions. Only the correlation coefficient of the Langmuir curve was lower. This fact suggests that heterogeneous surface conditions predominate, but monolayer biosorption may coexist. Accordingly, the biosorption process on the bacteria is complex, involving several mechanisms.

The maximum biosorption capacity (*q*_*m*_) was obtained with the Langmuir model (140.18 mg/g), which is comparable to other types of biomass studied (Table 9). A direct comparison between different biosorbents is difficult because of the varying experimental conditions employed. However, it can be seen as* Klebsiella* sp. 3S1 exhibits good biosorption efficiency, among the highest that have been reported for lead ions.

## 4. Conclusions

This work concluded that the isolate* Klebsiella* sp. 3S1 may be employed to be used as an inexpensive biosorbent, highly efficient for Pb(II) uptake. Maximum biosorption capacity (140.19 mg/g dry cell) was obtained at 25°C and an initial pH of 5. As a result of TEM-EDX analysis, both cell-surface binding and bioaccumulation could be involved in Pb removal. The biosorption process was found to be dependent on experimental factors such as the biosorbent dosage, initial metal ion concentration, pH, temperature, and contact time. Biosorption equilibrium data fit better to the Freundlich model, which implies heterogeneous surface conditions. This result is in accordance with the kinetic studies, which show that the sorption process is slow and that biosorption is performed in two phases: a very fast initial rate within the first 5 min, followed by slow attainment of equilibrium within 1 to 2 days, both of which contribute to the total metal biosorption. FTIR and SEM-EDX techniques confirmed the interactions between lead ions and functional groups on the wall surface of* Klebsiella* sp. 3S1. Moreover, TEM-EDX analysis showed the presence of metal in the cytoplasm.

Future research should be carried out to develop a robust immobilisation method for wastewater treatment, including continuous biosorption with reuse and recycling.

## Figures and Tables

**Table 1:** Experimental design and response.

Run order	Factor			Response
	*B*: biosorbent dosage (mg/mL)	pH	*T*: temperature (°C)	*q*_*e*_: biosorption capacity (mg/g)
12	0.55	5.50	25	104.02
6	0.80	4.30	34	118.51
13	0.55	4.75	10	80.97
11	0.55	4.00	25	87.42
1	0.30	4.30	16	67.27
15	0.55	4.75	25	105.02
18	0.55	4.75	25	105.75
4	0.80	5.20	16	86.16
10	1.00	4.75	25	94.99
19	0.55	4.75	25	108.65
5	0.30	4.30	34	127.21
9	0.10	4.75	25	122.13
3	0.30	5.20	16	76.36
7	0.30	5.20	34	138.52
16	0.55	4.75	25	105.31
2	0.80	4.30	16	79.17
20	0.55	4.75	25	106.18
8	0.80	5.20	34	119.79
14	0.55	4.75	40	163.53
17	0.55	4.75	25	106.18

**Table 2:** Kinetic models tested.

Model	Equation

Pseudo-first order or Lagergren [14]	$\frac{dq}{dt}=k_{1}\left(q_{e}-q\right)$
Pseudo-second order [15]	$\frac{dq}{dt}=k_{2}{\left(q_{e}-q\right)}^{2}$
Elovich [16]	$\frac{dq}{dt}=ae^{-bq}$
Intraparticle diffusion or Weber and Morris [14]	$q=k\sqrt{t}$

*q*: biosorption capacity (mg/g) at time *t*, according to (1).

*q*_*e*_: biosorption capacity (mg/g) at equilibrium.

*k*_1_: pseudo-first-order kinetic rate constant (min^−1^).

*k*_2_: pseudo-second-order kinetic rate constant (g·mg^−1^·min^−1^).

*a*: Elovich constant (mg·g^−1^·min^−1^).

*b*: Elovich constant (g·mg^−1^).

*k*: intraparticle diffusion rate constant in the bacterium (mg·g^−1^·min^−1/2^).

**Table 3:** Isotherm models used to represent the biosorption equilibrium.

Model	Equation
Langmuir [17]	$q_{e}=\frac{q_{m}bC_{e}}{1+bC_{e}}$
Freundlich [18]	*q*_*e*_ = *K*_*F*_ *C*_*e*_^1/*n*^
Sips [19]	$q_{e}=\frac{K_{s}C_{e}^{1/n}}{1+a_{s}C_{e}^{1/n}}$
Redlich-Peterson [20]	$q_{e}=\frac{K_{RP}C_{e}}{1+a_{RP}C_{e}^{\beta }}$

*q*_*e*_: biosorption capacity (mg/g) at equilibrium.

*q*_*m*_: maximum biosorption capacity (mg/g).

*b*: Langmuir biosorption equilibrium constant (L/mg).

*C*_*e*_: equilibrium concentrations of metal (mg/L).

*K*_*F*_: characteristic constant related to the biosorption capacity.

*n*: characteristic constant related to the biosorption intensity.

*K*_*s*_ and *a*_*s*_: Sips isotherm parameters.

*K*_RP_, *a*_RP_, and *β*: Redlich-Peterson parameters; and *β* varies between 0 and 1.

**Table 4:** Lead biosorption capacity from preliminary tests.

Isolates	*q* (mg/g)
Fungi	
*Galactomyces geotrichum *5L2	66.00
*Penicillium *sp. 8L2	44.74
*Pseudallescheria boydii *3S3	85.17
*Trichosporon *sp. 1L1	104.53
Yeasts	
*Trichosporon *sp. 4L2	22.29
*Trichosporon *sp. 4S3	35.72
*Rhodotorula mucilaginosa *1S1	23.95
*Rhodotorula mucilaginosa *2S4	37.59
Bacteria	
*Klebsiella *sp. 3S1	90.48
*Enterobacter *sp. 2E2	78.10

**Table 5:** IR absorption bands: changes and possible assignment.

FTIR peak	Original biomass wavenumbers (cm^−1^)	Pb(II) loaded biomass wavenumbers (cm^−1^)	Displacement (cm^−1^)	Functional groups	Assignment
1	3282	3280	2	–OH, –NH	Stretching vibrations of amino and hydroxyl groups
2	2958	2957	1	–CH_3_	–CH_3_ asymmetric stretching
3 4	2925 2875	2924 2875	1 0	–CH_2_ –CH_3_	–CH_2_ asymmetric stretching vibrations –CH_3_ symmetric stretching vibrations
5	2854	2853	1	–CH_2_	–CH_3 _asymmetric stretching vibrations
6	1638	1639	1	–CO, C–N	C=O and C–N stretching in amide I group
7	1531	1534	3	–CN, –NH	C–N stretching in amide II group and N–H bending
8	1453	1453	0	–CH_2_, –CO	–CH_2_ bending, symmetric C=O
9	1396	1398	2	–COO^−^	–COO^−^ symmetric stretching of carboxyl groups
10	1233	1236	3	–PO_2_^−^, –CO	P=O asymmetric stretching of phosphate groups, deformation vibration of C=O carboxylic acids
11 12	1063	1058 1038	5 1038	–PO_2_^−^, –OH –CO, PO_2_^−^	P=O symmetric stretching of phosphate groups, –OH of polysaccharides C–O stretching of alcoholic groups, symmetric stretching of phosphate groups
13		993	993	–C–O, –CH_2_	C–O–C, C–O–P, and –CH_2_ stretching vibrations of polysaccharides
14	967	969	2		N-containing bioligands
15	914	915	1		N-containing bioligands
16	860	861	1		S=O stretching
17	796	796	0		N-containing bioligands
18	780	780	0		N-containing bioligands
19		571	571		N-containing bioligands
20	515	539	24		N-containing bioligands

**Table 6:** Integrated equations, boundary conditions, and kinetic parameters of the biosorption by *Klebsiella *sp. 3S1.

		Exp. number 1	Exp. number 2
*Pseudo-first order *			
*q* = 0 at *t* = 0 and *q* = *q* at *t* = *t* *q* = *q*_*e*_(1 − *e* ^−*k*_1_*t*^)	*q*_*e*_	103.2	78.15
	*k*_1_	0.00775	0.00607
	*r* ^2^	0.437	0.627
	∑(*q* − *q*_cal_)^2^	5826	2781
*q* = *q*_*i*_ at *t* = 0 and *q* = *q* at *t* = *t* *q* = *q*_*e*_(1 − *e* ^−*k*_1_*t*^) + *q*_*i*_ *e* ^−*k*_1_*t*^	*q*_*e*_	115.7	85.43
	*q*_*i*_	49.43	30.21
	*k*_1_	0.0013	0.0018
	*r* ^2^	0.992	0.983
	∑(*q* − *q*_cal_)^2^	81.65	124.5

*Pseudo-second order *			
*q* = 0 at *t* = 0 and *q* = *q* at *t* = *t* $q=\frac{t}{1/k_{2}q_{e}^{2}+t/q_{e}}$	*q*_*e*_	107.5	82.64
	*k*_2_	1.53*E* − 04	1.40*E* − 04
	*r* ^2^	0.629	0.774
	∑(*q* − *q*_cal_)^2^	3836	1686
*q* = *q*_*i*_ at *t* = 0 and *q* = *q* at *t* = *t* $q=q_{e}-\frac{q_{e}-q_{i}}{1+k_{2}t\left(q_{e}-q_{i}\right)}$	*q*_*e*_	130.9	96.56
	*q*_*i*_	47.57	28.84
	*k*_2_	1.75*E* − 05	3.00*E* − 05
	*r* ^2^	0.996	0.974
	∑(*q* − *q*_cal_)^2^	45.26	197.4

*Elovich *			
*q* = 0 at *t* = 0 and *q* = *q* at *t* = *t* $q=\frac{1}{b}\ln\left(1+abt\right)$	*a*	26.55	7.176
	*b*	0.07855	0.08845
	*r* ^2^	0.922	0.923
	∑(*q* − *q*_cal_)^2^	811.1	570.1
*q* = *q*_*i*_ at *t* = 0 and *q* = *q* at *t* = *t* $q=\frac{1}{b}\ln\left(abt+e^{bq_{i}}\right)$	*a*	1.832	1.516
	*b*	0.04807	0.06404
	*q*_*i*_	45.76	26.39
	*r* ^2^	0.992	0.962
	∑(*q* − *q*_cal_)^2^	78.03	286.7

*Intraparticle diffusion *			
$q=k\sqrt{t}$	*k*	2.488	1.971
	*r* ^2^	—	0.241
	∑(*q* − *q*_cal_)^2^	11718	5652
$q=q_{i}+k\sqrt{t}$	*k*	1.305	1.146
	*q*_*i*_	48.84	31.50
	*r* ^2^	0.957	0.903
	∑(*q* − *q*_cal_)^2^	439.8	719.0

Exp. number 1: 50 mg/L of Pb(II) and 0.11 g/L of dry biomass. Exp. number 2: 25 mg/L of Pb(II) and 0.28 g/L of dry biomass. *r* ^2^ is the correlation coefficient, and ∑(*q* − *q*_cal_)^2^ is the sum of the errors squared.

**Table 7:** ANOVA for the response surface reduced quadratic model.

Source	Sum of squares	DF	Mean square	*F*-value	*p* value
Model	9588.53	8	1198.57	437.66	<0.0001
*B*: biosorbent dosage	4.10	1	4.10	1.50	0.2520
pH	234.13	1	234.13	85.49	<0.0001
*T*: temperature	8164.13	1	8164.13	2981.14	<0.0001
*B*pH	18.39	1	18.39	6.72	0.0291
*BT*	301.72	1	301.72	110.17	<0.0001
*B* ^2^	161.57	1	161.57	59.00	<0.0001
pH^2^	164.17	1	164.17	59.95	<0.0001
*T* ^2^	387.29	1	387.29	141.42	<0.0001
Residual	24.65	9	2.74		
Lack of fit	16.26	4	4.06	2.42	0.1790
Pure error	8.39	5	1.68		
Cor total	9613.18	17			

CV %	1.58				
*R*-Squared	0.9974				
Adj. *R*-Squared	0.9952				
Pred. *R*-Squared	0.9837				

**Table 8:** Biosorption equilibrium parameters of the isotherm models by *Klebsiella *sp. 3S1.

Langmuir	*q*_*m*_	140.19
	*b*	0.075353
	*r* ^2^	0.9395
	∑(*q* − *q*_cal_)^2^	60.955

Freundlich	*K*_*F*_	65.266
	*n*	7.4312
	*r* ^2^	0.9901
	∑(*q* − *q*_cal_)^2^	15.920

Sips	*K*_*s*_	66.223
	*a*_*s*_	0.019748
	*n*	7.1713
	*r* ^2^	0.9901
	∑(*q* − *q*_cal_)^2^	15.915

Redlich-Peterson	*K*_RP_	20850.6
	*a*_RP_	319.3393
	*β*	0.86949
	*r* ^2^	0.9901
	∑(*q* − *q*_cal_)^2^	15.923

*r* ^2^ is the correlation coefficient.

∑(*q* − *q*_cal_)^2^ is the sum of the errors squared.

**Table 9:** Maximum lead biosorption capacity of different microorganisms.

Biosorbent	*q*_*m*_ (mg/g)	Reference
*Penicillium* sp.	60.77	[21]
*R. arrhizus *	48.79	[22]
*Ceratophyllum demersum *	44.80	[23]
*Bacillus cereus *M_16_^1^	70.42	[24]
*Aspergillus niger *	34.92	[25]
Dried activated sludge	131.60	[26]
*Caulerpa lentillifera *	28.99	[27]
*Cladophora fascicularis *	227.70	[28]
Immobilised *Saccharomyces cerevisiae *	30.04	[29]
Recombinant *Escherichia coli *	108.99	[30]
*Klebsiella *sp. 3S1	140.19	In this study
